# Indonesian herbal medicine prevents hypertension-induced left ventricular hypertrophy by diminishing NADPH oxidase-dependent oxidative stress

**DOI:** 10.18632/oncotarget.21424

**Published:** 2017-09-30

**Authors:** Erna Sulistyowati, Jong-Hau Hsu, Yuan-Bin Cheng, Fang-Rong Chang, Ying-Fu Chen, Jwu-Lai Yeh

**Affiliations:** ^1^ Graduate Institute of Medicine, College of Medicine, Kaohsiung Medical University, Kaohsiung, Taiwan; ^2^ Faculty of Medicine, Islamic University of Malang, Malang, East Java Province, Indonesia; ^3^ Department of Pediatrics, College of Medicine, Kaohsiung Medical University, Kaohsiung, Taiwan; ^4^ Department of Pediatrics, Kaohsiung Medical University Hospital, Kaohsiung, Taiwan; ^5^ Graduate Institute of Natural Products, College of Pharmacy, Kaohsiung, Taiwan; ^6^ Division of Cardiovascular Surgery, Department of Surgery, Kaohsiung Medical University Hospital, Kaohsiung, Taiwan; ^7^ Sin-Lau Christian Hospital, Tainan, Taiwan; ^8^ Department of Pharmacology, College of Medicine, Kaohsiung Medical University, Kaohsiung, Taiwan; ^9^ Department of Medical Research, Kaohsiung Medical University Hospital, Kaohsiung, Taiwan; ^10^ Department of Marine Biotechnology and Resources, National Sun Yat-Sen University, Kaohsiung, Taiwan

**Keywords:** Indonesian traditional medicine, hypertension, oxidative stress, NADPH oxidase

## Abstract

Indonesian herbal medicine *Centella asiatica*, *Justicia gendarussa* and *Imperata cylindrica* decoction (CJID) are known to be efficacious for hypertension. Oxidative stress plays an important role in hypertension-induced left ventricular hypertrophy (H-LVH). This study evaluated whether CJID inhibit cardiac remodeling in spontaneously hypertensive rats (SHRs) through mechanism of oxidative stress-related cardiac-NADPH oxidase (NOXs) pathway: NOX1, NOX2 and NOX4. Forty-weeks-old SHRs and normotensive-WKY rats, were both randomly divided into 2 groups: CJID and control. All rats were treated for 5 weeks. Systolic blood pressure (SBP) and heart rate (HR) were measured. LV morphology, function and performance were assessed by histological staining and echocardiography. Serum and cardiac superoxide dismutase (SOD) activity and malondialdehyde (MDA) content were assessed. Cardiac superoxide and hydrogen peroxide (H_2_O_2_) productions, protein expressions of SOD2, SOD3, NOX1, NOX2 and NOX4 were also determined. We found that SBP and HR were significantly decreased in SHRs-treated group. Echocardiography showed that CJID significantly improved LV morphometry and function. CJID decreased MDA level, but increased SOD activity. Cardiac superoxide and H_2_O_2_ generation were decreased in SHRs-treated group. CJID caused cardiac SODs expressions to be increased but NOXs expressions to be suppressed. In conclusion, CJID prevents H-LVH by reducing reactive oxygen species production via the NOXs-dependent pathway.

## INTRODUCTION

Hypertension sets to become a vital factor in health worldwide, since it causes increase in death rate and disability among people in many countries [1]. It is evident that hypertension is identified as a concomitant risk for cardiovascular disease. A well-recognized risk of cardiovascular-related death is left ventricular hypertrophy (LVH) [2]. The mechanism driving hypertensive LVH reveals various key factors, including hemodynamic load, endothelial, neuro-humoral and oxidative stress. Hypertension is characterized by elevation of arterial blood pressure (BP), and subsequently affects structural and functional changes in the left cardiac ventricle. Chronic pressure results in the hypertrophic adaptation of existing cardiomyocytes with increased width, followed by thickening of left ventricular wall [3–5]. Accordingly, LVH is a sensitive indicator of early alteration in the heart because of pressure overload in hypertension [6, 7]. Furthermore, it is an important risk factor of coronary heart disease, heart failure and stroke in patients with hypertension [3, 8]. Indeed, recent studies have shown that LVH can increase cardiac-related death [9]. Therefore, the improvement of cardiac performance can be reached by inhibition of LVH independently of other risk factors [10].

NADPH oxidase (NOX) has an essential role in the development of cardiovascular disease such as atherosclerosis, hypertension, cardiac hypertrophy and remodeling, angiogenesis and collateral formation, stroke and heart failure [11]. NOX enzymes are expressed in virtually all cardiovascular tissue and they regulate diverse functions such as differentiation, proliferation, apoptosis, senescence, inflammatory responses and oxygen sensing [12]. In hypertension, NOX is implicated in the increase in oxidative stress through excessive generation of reactive oxygen species (ROS) [13]. Recent study suggests the role of certain dominant NOX family such as NOX1, NOX2, and NOX4 in contributing ROS-induced hypertensive LVH [14]. Therefore, targeting cardiac NOX as a strategy of inhibiting hypertensive LVH has received much attention in the past decade [15].

Recently, herb-based traditional medicine has been widely used and is rapidly growing among many countries, and World Health Organization has encouraged the utilization of natural medicine as potential therapeutic strategy [16]. Indonesia has many indigenous medicinal plants which Indonesian rural communities have been using to treat diseases for several centuries. Among these plants, *jamu*, a word of Java tribal language, is subject to traditional herbal medicine [17]. Currently, a well-known potential anti-hypertensive formula of *jamu*, containing celery leaves (*Apium graviolens*) and cat-whisker leaves (*Orthosiphon stamineus benth*), has been used for years after it was designed as a new phytopharmaca [18]. Moreover, another anti-hypertensive herb, gotu kola leaves (*Centella asiatica*), was also used to treat hypertension [19]. By contrast, based on the reasons concerning conservation circle and sustainability of plant resources [20], it is truly crucial to develop an alternative anti-hypertensive formula. One other herbal anti-hypertensive formula consisted of *Centella asiatica*, willow-leaved justicia (*Justicia gendarussa*) and cogon grass (*Imperata cylindrica*), decoction extraction method (CJID). Although CJI decoction has been known and widely used in treating hypertension both in formal healthcare services and by indigenous people in Indonesia, the mechanisms of its effects remain unclear. In addition, there has been no data regarding its role in cardiac protection. However, its anti-hypertrophic effects and mechanisms in left ventricle are still unknown. Thus, this study is to examine if CJID can inhibit LVH through the NOX-dependent pathway.

## RESULTS

### Effects of CJID on SBP

**Figure 1:**
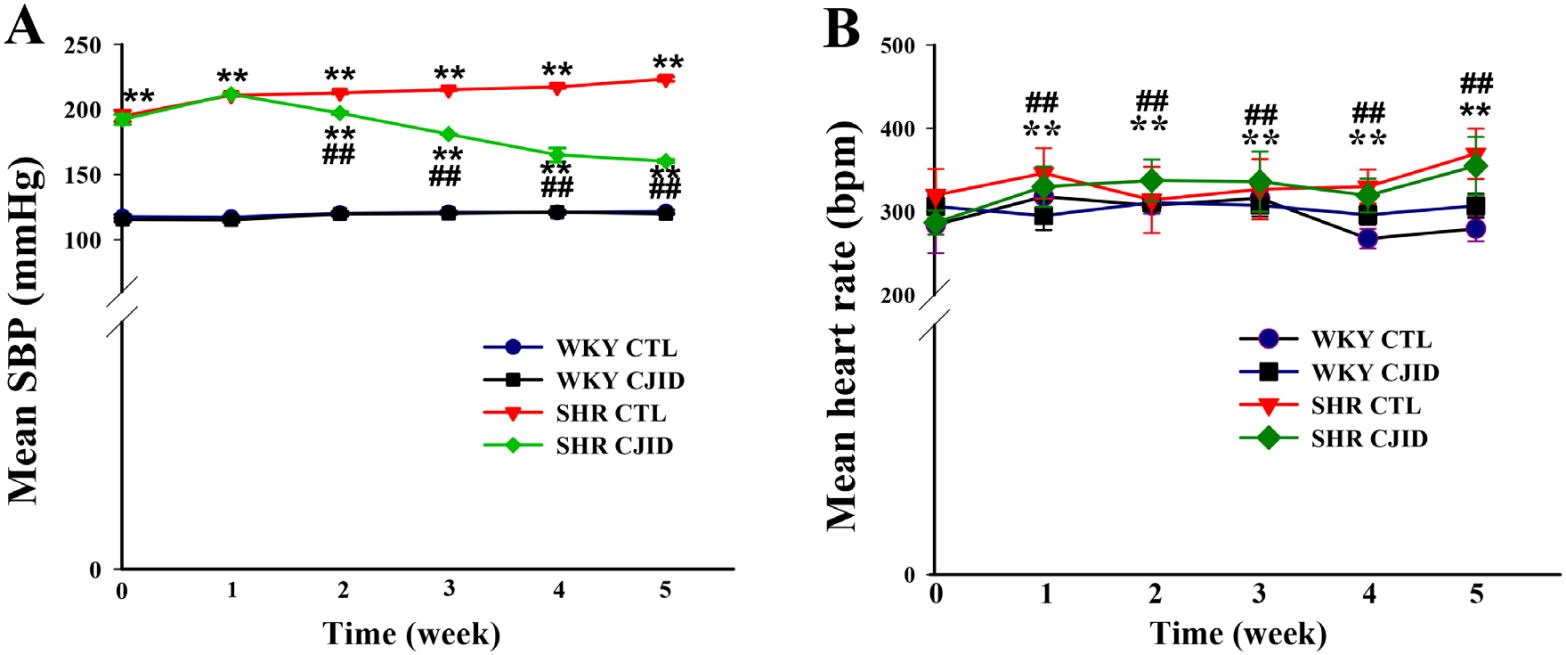
Effects of CJI decoction on mean SBP and mean HR evolution The observation were five weeks of study (6 times). **(A)** The SBP evolution and **(B)** the HR evolution. Each point represents the mean ± SEM, *n* = 10. **P < 0.01, *when compared to WKY CTL; ^##^*P* < 0.01, ^#^when compared to SHR CTL.

Figure 1A shows the initial mean of SBP was significantly higher in the naive SHRs than in the WKY rats (*P* < 0.01). After treatment for 2 weeks, SBP was significantly lower in SHRs and it continued to decrease in subsequent weeks by 6.7%, 6.8%, 22% and 24.2%, respectively until end of study (*P* < 0.01). The final SBP mean was 160.3 ± 1.1 mmHg (*P* < 0.01) in SHRs compared with its control (223.3 ± 1.7 mmHg) (Table 1).

**Table 1:** Systolic blood pressure and heart rate at the end of five weeks

	Mean SBP (mmHg)		Mean HR (bpm)	
Group	Before	After	Before	After
**WKY CTL**	117.3 ± 0.7	121.5 ± 1.3	295.3 ± 15.5	279.1 ± 15.1
**WKY CJID**	115.1 ± 1.5	120.0 ± 0.4	303.6 ± 13.4	306.8 ± 14.7
**SHR CTL**	210.8 ± 0.7^**^	223.3 ± 1.7^**^	334.2 ± 31.4	398.2 ± 10.5^**^
**SHR CJID**	211.6 ± 0.7^**^	160.3 ± 1.1^**##^	327.2 ± 25.8	355.7 ± 15.6^*#^

Each point represents the mean ± SEM, *n* = 10. *P < 0.05, **P < 0.01, * when compared to WKY CTL; ^#^*P* < 0.05, ^##^*P* < 0.01, ^#^when compared to SHR CTL.

### Effects of CJID on HR

As shown in Figure 1B, HR evolution was significantly lower after 5 weeks in both WKY groups (*P* < 0.01) than in control group of SHRs. CJID treatment lowered HR in SHR, which started from second week until fifth week (*P* < 0.01) (Table 1). The increase of SBP and HR in SHRs cause subsequent cardiac damage. Therefore, we proceeded to investigate whether CJID prevented cardiac remodeling.

### CJID alleviated left ventricular hypertrophy in SHRs

**Figure 2:**
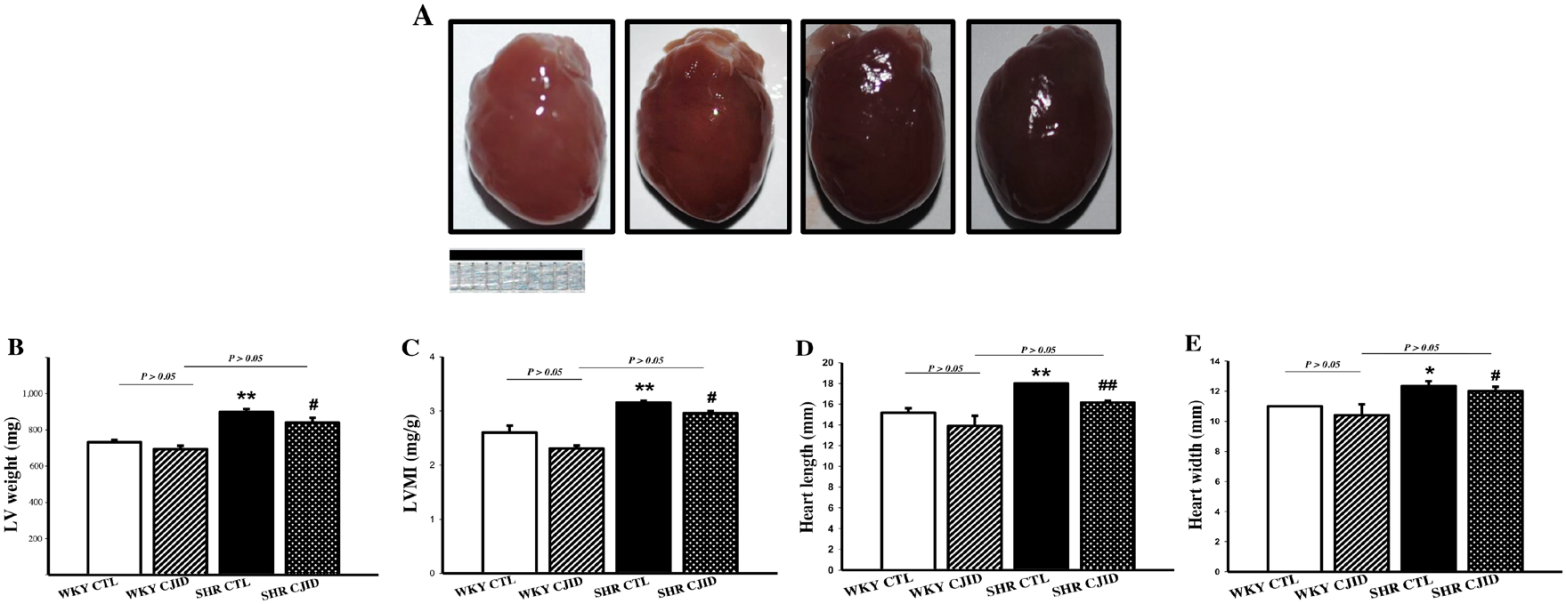
Effect of CJID on the cardiac morphology **(A)** Heart pictures, **(B)** left ventricular weight (mg), **(C)** LVMI, **(D)** heart length (mm), and **(E)** heart width (mm). Each point represents the mean ± SEM, *n* = 10. *P < 0.05, **P < 0.01, *when compared to WKY CTL; ^#^*P* < 0.05, ^##^*P* < 0.01, ^#^when compared to SHR CTL. Bar shows 10 mm.

**Figure 3:**
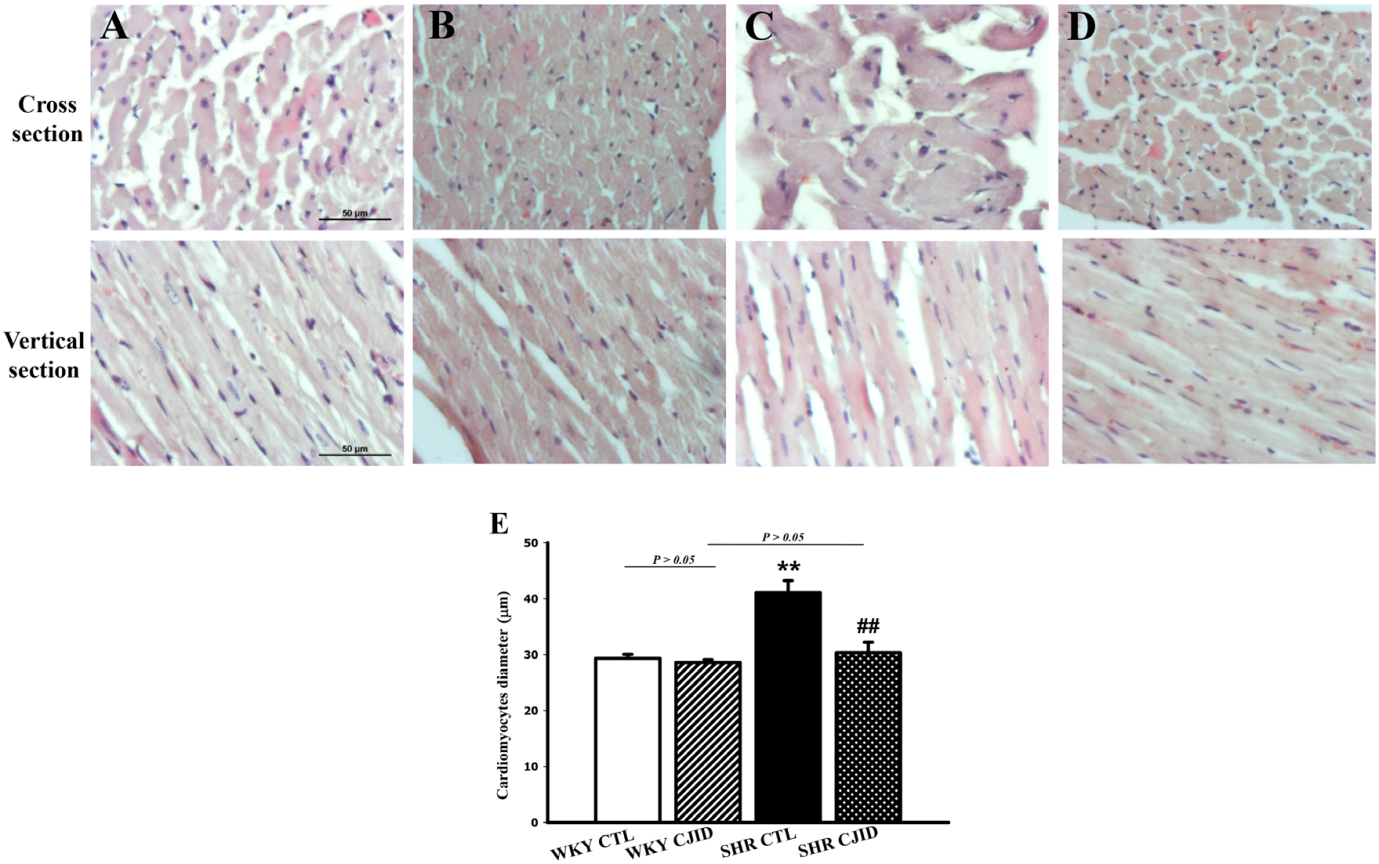
Microscopic observations (cross and vertical section; 400x) of myocardial tissue After 5 weeks treatment CJI decoction, the left ventricular myocardium from 4 groups including **(A)** the WKY CTL group, **(B)** the WKY CJID group, **(C)** the SHR CTL, and **(D)** the SHR CJID group was examined by haematoxylin and eosin. **(E)** The cardiomyocytes was measured the diameter (μm). Each point represents the mean ± SEM, *n* = 10. **P < 0.01, *when compared to WKY CTL; ^##^*P* < 0.01, ^#^when compared to SHR CTL.

As shown in Figure 2B, the SHR CTL has the largest left ventricular weight (LVW) compared with WKY groups (*P* < 0.01), while SHR CJID showed decrease in LVW (*P* < 0.05). In order to show the case of left ventricular hypertrophy, left ventricular mass index (LVMI) was measured. As presented in Figure 2C, SHR CTL had the highest LVMI, suggesting that left ventricular hypertrophic was present in SHRs (*P* < 0.01). In contrast, the SHR CJID group had significantly lower LVMI (*P* < 0.05). The heart length (Figure 2D) and the width (Figure 2E) showed that SHRs had cardiac enlargement compared with WKY rats. By contrast, SHR CJID had significant smaller size than the SHR CTL (*P* < 0.05). As presented in Figure 3, the histologic examination showed that SHR CTL (C) had the largest cardiomyocytes diameter compared with WKY CTL (A, *P* < 0.01) and SHR CJID (D, *P* < 0.01). As also shown in gross heart images, Figure 2A, the LVMI, cardiac length and width measurement and histologic examination showed significant increase in SHR CTL group reflecting cardiac enlargement. By morphological examination, it definitely denoted a cardiac hypertrophy in SHR CTL group. CJID administration inhibited LVH in SHRs.

### CJID inhibited left ventricular dysfunction in SHRs

**Figure 4:**
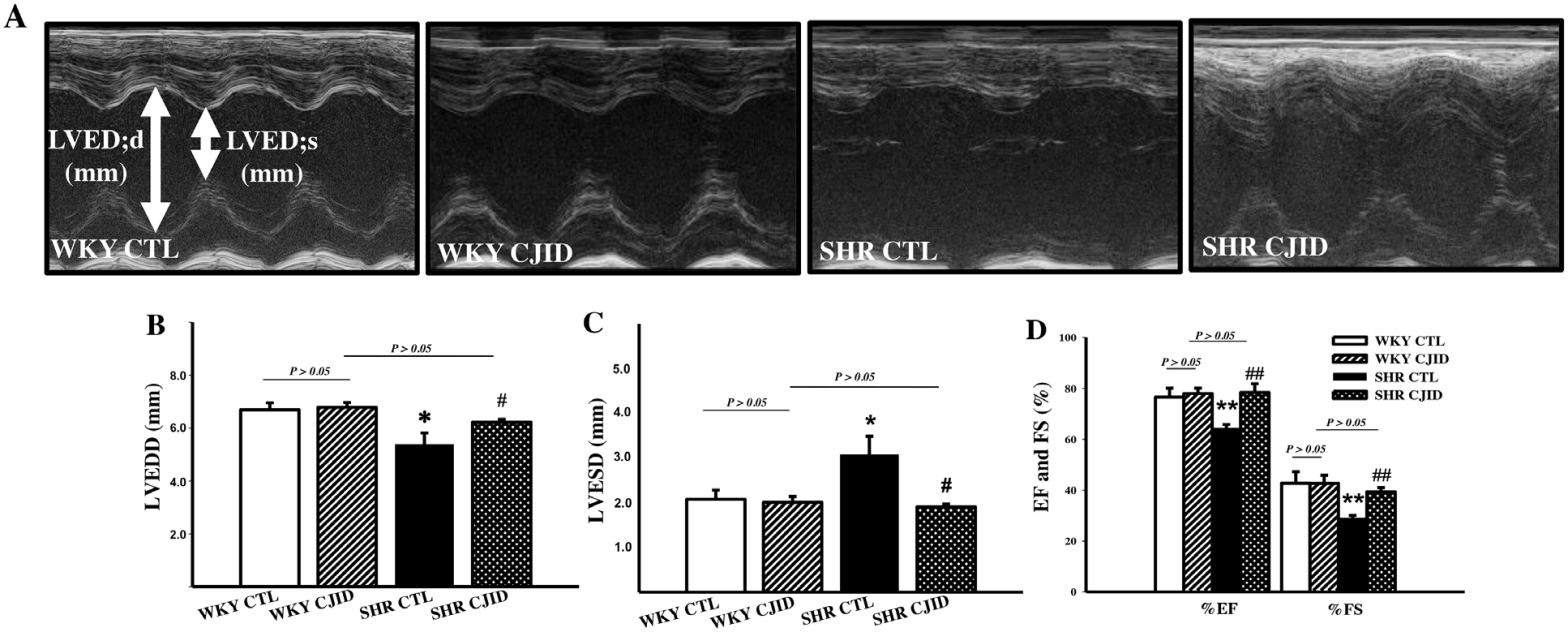
Effect of CJI decoction in the left ventricular function Measurement has been taken by echocardiography. **(A)** M-Mode echocardiogram images were represented from individual groups. **(B)** The values of LVEDD (left ventricular end-diastolic dimension), and **(C)** LVESD (left ventricular end-systolic dimension), **(D)** the percentage of FS (fractional shortening), and EF (ejection fraction) were calculated by echocardiographic parameters. Each point represents the mean ± SEM, *n* = 10. *P < 0.05, **P < 0.01, *when compared to WKY CTL; ^#^*P* < 0.05, ^##^*P* < 0.01, ^#^when compared to SHR CTL.

As shown in Figure 4B and 4C, the SHR CTL exhibited significant decrease value of left ventricular end-diastolic dimension (LVEDD) and increase value in left ventricular end-systolic dimension (LVESD, P < 0.05) compared with WKY CTL. There were no statistical difference in LVEDD and LVESD among WKY CTL, WKY CJID and SHR CJID. The lowest values of left ventricular fractional shortening (FS) and ejection fraction (EF) were found in SHR CTL (*P* < 0.01) compared with both WKY groups. CJID increased FS and EF values (*P* < 0.01). These results indicated that CJID improved left ventricular function in SHRs. M-mode echocardiogram images were presented on Figure 4A.

### CJID decreased MDA activity and increased SOD activity levels in SHRs

**Figure 5:**
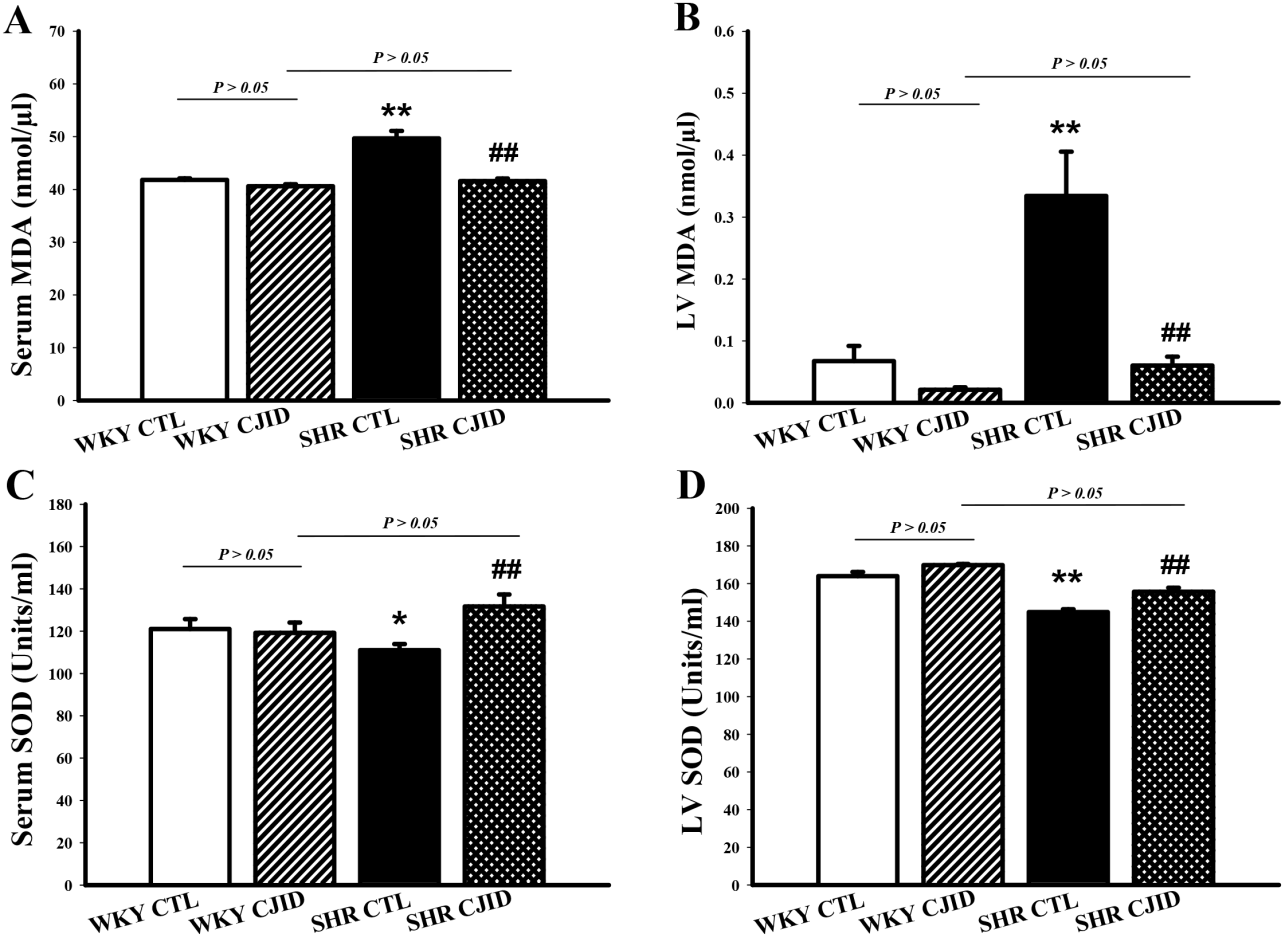
Effects of CJI decoction on the serum and left ventricular malondialdehyde and superoxide dismutase activity levels **(A)** The serum MDA activity levels of the 4 groups. **(B)** The left ventricular myocardium MDA activity levels of the 4 groups. **(C)** The serum SOD activity levels of the 4 groups. **(D)** The left ventricular myocardium SOD activity levels of the 4 groups. Each point represents the mean ± SEM, *n* = 10. *P < 0.05, **P < 0.01, *when compared to WKY CTL; ^##^*P* < 0.01, ^#^when compared to SHR CTL.

The serum and cardiac levels of MDA activities were significantly lower after CJID treatment in SHRs (*P* < 0.01). The serum and cardiac SOD activity levels were significantly increased in SHRs CJID-treated group (*P* < 0.01), while there was no significant difference in the other groups (Figure 5).

### CJID reduced fluorescence level of oxidative stress in left ventricular cardiomyocytes

**Figure 6:**
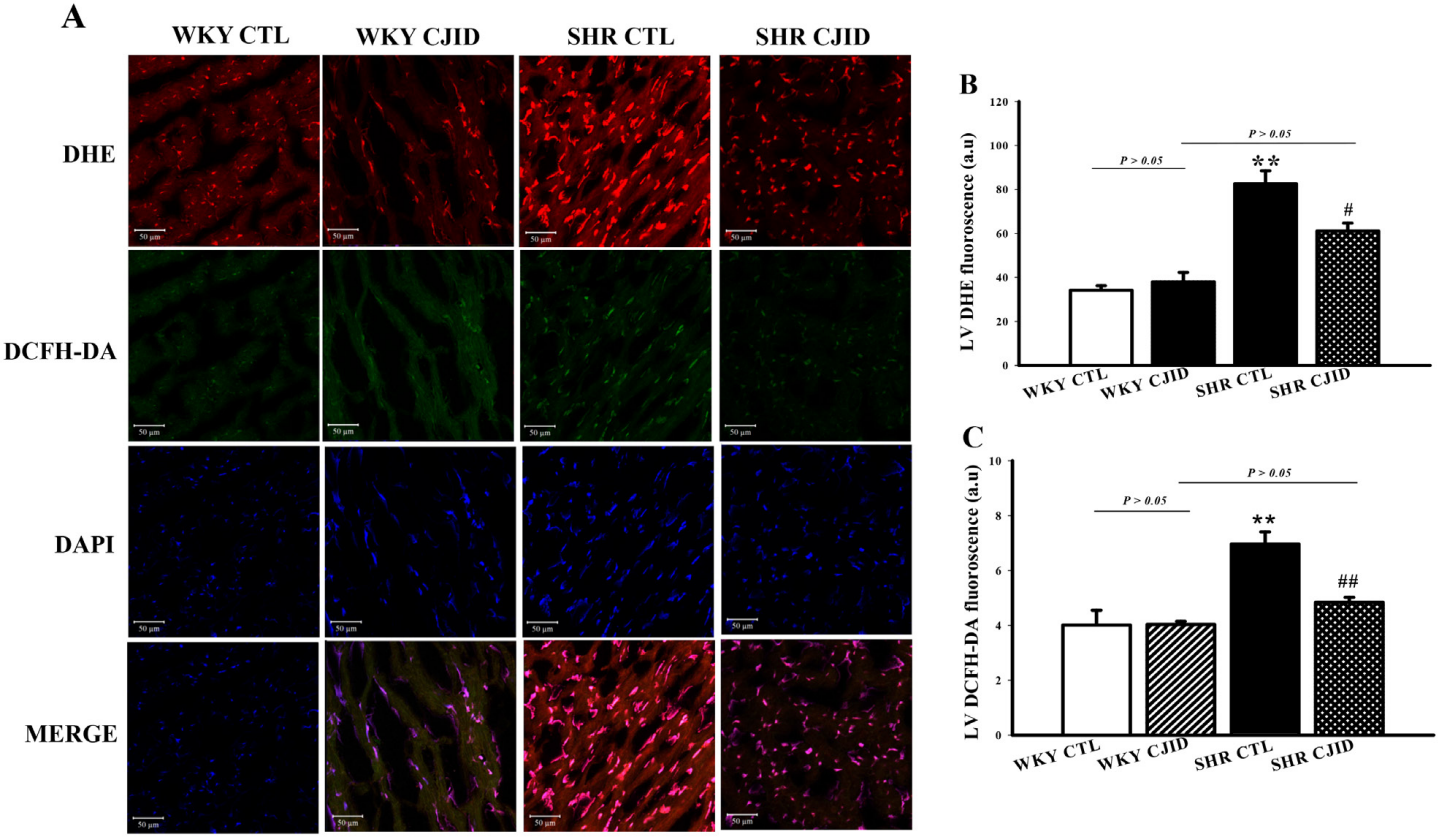
Confocal microscopic observations (200x) of nuclear cardiomyocytes After 5 weeks treatment of CJI decoction. **(A)** Anion superoxide was detected by DHE staining and H_2_O_2_ was detected by DCFH-DA staining from 4 groups including WKY CTL, WKY CJID, SHR CTL, SHR CJID, **(B)** DHE fluorescence of cardiomyocytes, and **(C)** DCFH-DA fluorescence of cardiomyocytes. Each point represents the mean ± SEM, *n* = 10. **P < 0.01, *when compared to WKY CTL; ^#^*P* < 0.05, ^##^*P* < 0.01, ^#^when compared to SHR CTL.

To investigate oxidative stress marker, we measured *in situ* cardiomyocytes superoxide generation by dihydroethidium (DHE) staining, and H_2_O_2_ production by 2′, 7′-dichlorodihydrofluorescein diacetate (DCFHDA) staining. As shown in Figure 6, superoxide generation in cardiomyocytes was 34.1 ± 2.1 a.u (arbitrary units), 38 ± 4.2 a.u, 82.6 ±5.9 a.u, and 61.1 ± 3.6 a.u, respectively in WKY CTL, WKY CJID, SHR CTL and SHR CJID. SHRs had significantly increased DHE fluorescence level than in WKY rats (*P* < 0.01). CJID treatment decreased superoxide production in SHRs (*P* < 0.05). The DCFHDA staining in cardiomyocytes was 4 ± 0.54 a.u, 4.03 ± 0.12 a.u, 6.97 ± 0.44 a.u, and 4.84 ± 0.18 a.u, respectively in WKY CTL, WKY CJID, SHR CTL and SHR CJID. SHRs had significantly increased DCFHDA fluorescence level than in WKY rats (*P* < 0.01). CJID treatment decreased H_2_O_2_ production in SHRs (*P* < 0.01).

### CJID led endogenous antioxidant SOD2 and SOD3 expressions to increase in left heart ventricle in SHRs

**Figure 7:**
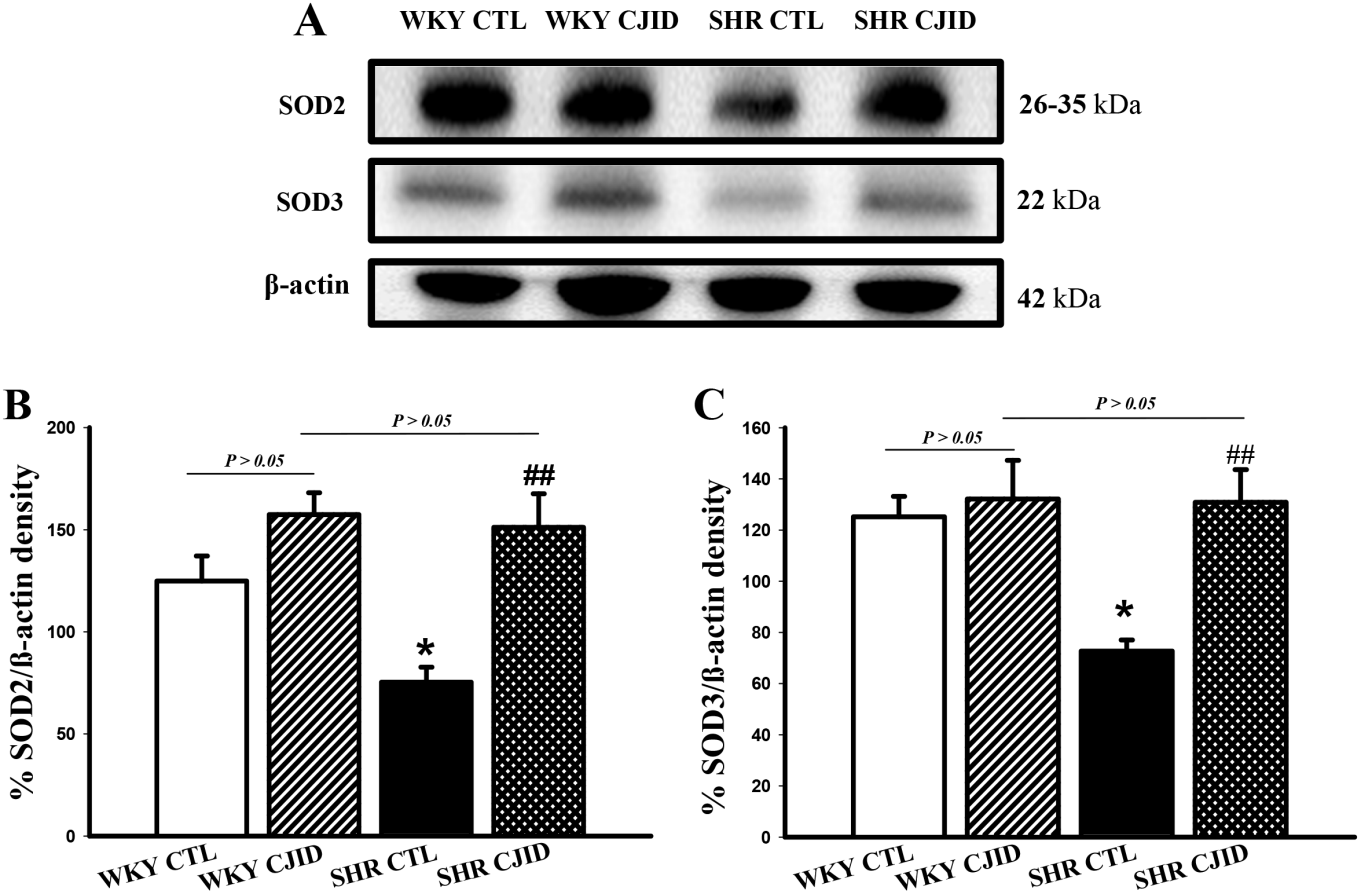
Effects of CJI decoction on the proteins expression levels of SOD2 and SOD3 in the left ventricular areas by western blotting analysis **(A)** The expression level of SOD2 and SOD3 in the left ventricular myocardium of the 4 groups. **(B)** The expression level of SOD2 in the left ventricular myocardium of the 4 groups. **(C)** The expression level SOD3 in the left ventricular myocardium of the 4 groups. Each point represents the mean ± SEM, *n* = 10. *P < 0.05, *when compared to WKY CTL; ^##^*P* < 0.01, ^#^when compared to SHR CTL.

To observe the impact on endogenous antioxidant activity, we evaluated the expressions of SOD2 and SOD3 in left heart ventricle. As presented in Figure 7A-7C, both SODs were decreased in their expressions in SHRs (*P* < 0.01). The treatment of CJID caused both SODs expressions to significantly increase in SHRs (*P* < 0.05).

### CJID decreased left heart ventricular NOX1, NOX2 and NOX4 expressions in SHRs

**Figure 8:**
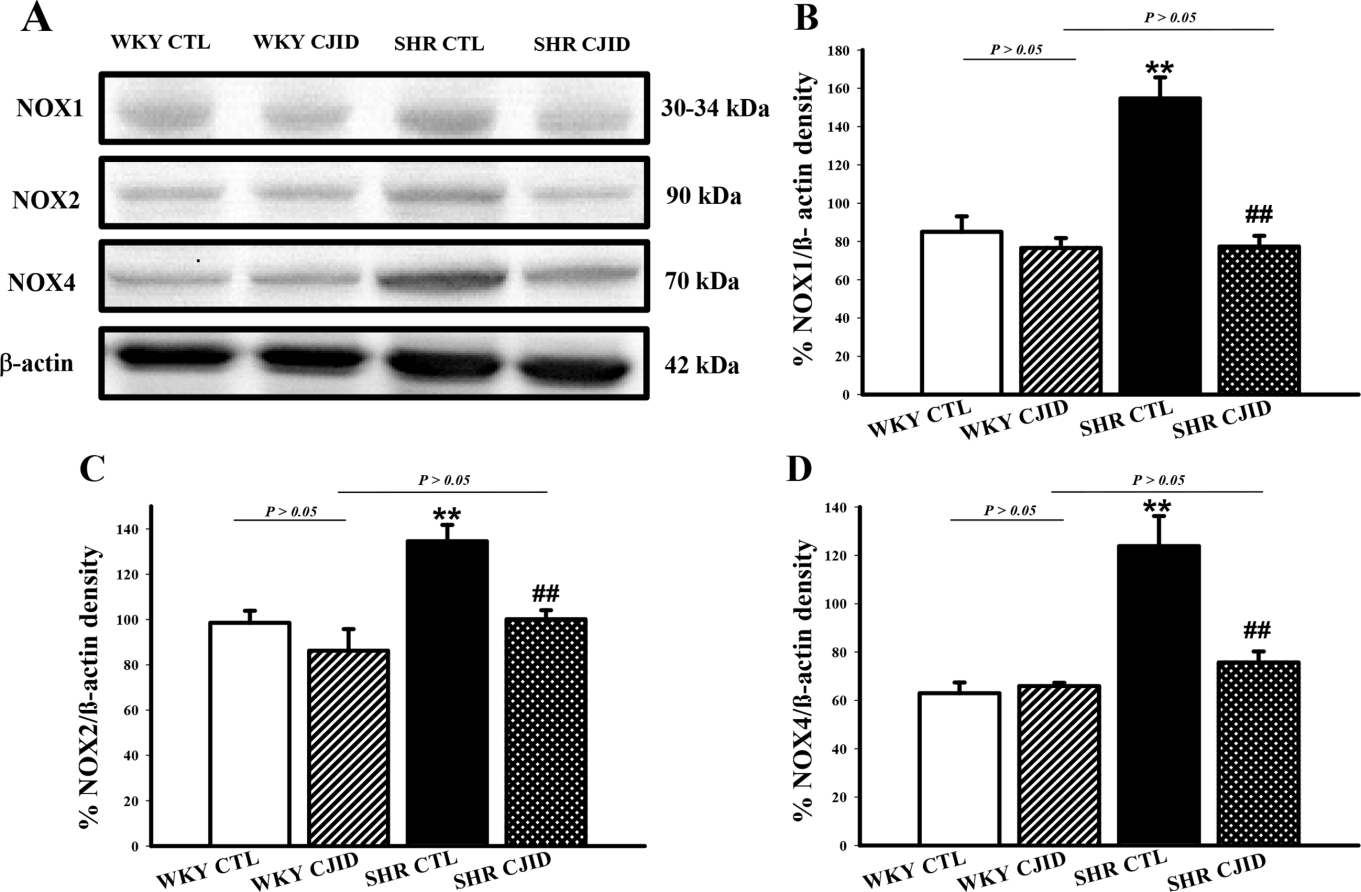
Effects of CJI decoction on the proteins expression levels of NOX1, NOX2 and NOX4 in the left ventricular areas by western blotting analysis **(A)** The expression levels of NOX1, NOX2 and NOX4 in the left ventricular myocardium of the 4 groups. **(B)** The expression level of NOX1 in the left ventricular myocardium of the 4 groups. **(C)** The expression level NOX2 in the left ventricular myocardium of the 4 groups. **(D)** The expression level NOX4 in the left ventricular myocardium of the 4 groups. Each point represents the mean ± SEM, *n* = 10. **P < 0.01, * when compared to WKY CTL; ^##^*P* < 0.01, ^#^when compared to SHR CTL.

To investigate the mechanism of decrease in oxidative stress, we observed the expressions of NADPH oxidases in cardiac left ventricle. As shown in Figure 8, the expressions of NOX1, NOX2 and NOX4 increased in SHRs. CJID treatment significantly decreased the expressions of cardiac NOX1, NOX2 and NOX4 in SHRs (*P* < 0.01).

## DISCUSSION

*Centella asiatica*, *Justicia gendarussa* and *Imperata cylindrica* have been in use traditionally since times immemorial to treat wide range of indications. They have been subjected to quite extensive phytochemical, experimental and clinical investigations. Their active constituents include vitamin C and polyphenolic constituents (flavonoids, triterpenoids, saponins and lignans). Experimental studies have demonstrated their functions in inflammation and antibacterial effect, wound healing activity, cytotoxic activity, neuroprotective effect, hepatoprotective effect, cardioprotective effect and oxidative stress inhibition activity (Table 2). In fact, traditional healers in Indonesia have been using these three herbs as an anti-hypertension from a very long time ago even though their scientific knowledge was limited. Therefore, this current study explores the therapeutic potential of these herbs in order to establish them as a standard drug.

**Table 2:** Composition of CJI decoction

English name	Latin name	Local name	Collecting time	Part used	Amount	Main chemical components	Pharmacological activity	Ref
Penny-wort, gotu kola, pegaga	*Centella asiatica*	Pegagan (Bahasa), Peugaga (Aceh), jalukap (Banjar), daun kaki kuda (Melayu), ampagaga (Batak), antanan, dulang sontak(Sunda), gagan-gagan, rendeng, cowek-cowekan, pane gowang (Jawa), piduh (Bali), bebele (Lombok), sandanan (Irian) broken copper coin, semanggen (Indramayu, Cirebon), pagaga (Makassar), daun tungke (Bugis), Pigago (Minang) daun tapak kudo (solok)	All seasons	leaf	5 g	Triterpenoids (asiaticoside, asiatic acid, and madecassic acid), flavonoids (quercetin, kaempferol and astragalin)	Mean arterial blood pressure (MABP) ↓, SBP↓, diastolic blood pressure (DBP) ↓ in L-NAME-induced hypertension rat, mPAP ↓, improving RVH and medial wall thickness in HPH (TGF-β1 Smad2/3 signaling), lowering venous hypertension and improving glucose& lipid levels in human-subjected study, insulin sensitivity ↑, improving lipid profiles and hemodynamic parameters, oxidative stress markers ↓, plasma TNF-α ↓, NOX ↓, and eNOS/iNOS expressions ↓ in MS rats, cardioprotective effects in LPS-mediated sepsis (blocking of ERK1/2, p38 and NF-κB pathways).	[43–45]
Willow-leaved justicia	*Justicia gendarussa*	Besi-besi (Aceh), gandarusa (Melayu), handarusa (Sunda), tetean, trus (Jawa), ghandharusa (Madura), gandarisa (Bima), puli (Ternate).	All seasons	leaf	5 g	Ascorbic acid, flavonoids (quercetin, kaempferol, astragalin, apigenin and vitexin)	Free radicals scavenging activity (O_2_^∙−^, H_2_O_2_) ↓, lipid peroxidation ↓, inhibit breast cells cancer, inhibit iNOS and COX-2 expression via NF-κB pathway, antiinflammation, antibacterial, antiarthritic.	[46, 47]
Cogon grass	*Imperata cylindrica*	alang-alang	All seasons	Stem and rhizome	3 g	Lignans (*Graminone B*), Triterpenoids (asiaticoside), flavonoids (quercetin, kaempferol and astragalin)	Contraction of jejunum smooth muscle cells in rabbit ↓, heart contraction ↓ in cats, vasodilator, antihypertensive diuretic, antiplatelet aggregating activity, inhibit NOS.	[48–50]

Abbreviation: ↑: up regulation; ↓: down regulation; mPAP: mean pulmonary artery pressure; HPH: hypoxia-induced pulmonary hypertension; RVH: right ventricular hypertrophy; MS: metabolic syndrome; iNOS: inducible nitric oxide synthase; eNOS: endothelial nitric oxide synthase; COX: cyclooxygenase.

Clinical trials have confirmed that consumption of fruits and vegetables rich in bioactive compounds including vitamins and polyphenols have demonstrated to protect against oxidative stress which reduces the risk of cardiovascular outcomes [21, 22]. Recent studies on DASH (Dietary Approaches to Stop Hypertension) recommend higher scores of DASH decrease cardiovascular risk [23]. That is, on the basis of available evidence, the use of herbs rich in bioactive compounds will well-establish antihypertensive activity in humans, which could represent a good compromise for treating hypertension with cardiac remodeling. In our experiment, a significant reduction of SBP by CJID was identified on the second week of treatment. Although there are differences between SHRs and hypertensive patients, the antihypertensive effect of CJID was partially reflected by the variation of BP. Biochemically, the pharmacological mechanism of CJID is closely related to the major active compounds including triterpenoids (asiaticoside, asiatic acid, madecassic acid and lupeol), flavonoids (quercetin, kaempferol and astragalin), lignans (*Graminone B*), and ascorbic acid. In spite of the fact that environmental factors, such as the site of cultivation, altitude, temperature, sun exposure time, rainfall, climate, and soil can influence the primary and secondary metabolites of herbs [24], so their bioactivities could be varied [25]. As predicted, our experiment showed that high levels of phenolic compounds in CJID and vitamin C derived from *J. gendarussa* decoction are responsible in this fashion. Thus, this study provided evidence that CJID could be used to lower BP and is beneficial for hypertension treatment. To determine that CJID functioned in the state of hypertension, we constructed normotensive group (WKY) treated with CJID. Our data revealed that CJID reduced SBP only in SHR, but not in WKY rats. We can hypothesized that the treatment likely counteract the misregulated biochemical pathways associated with hypertension. The lowering of SBP activity persisted until fourth week of study, and there was no more decrease in SBP up to fifth week. It implied that the treatment had reached the initial BP target.

Recently, it has been suggested that autonomic nervous system have fundamental function in controlling BP and development of hypertension [26]. An elevated resting HR is a predictor of increased risk of cardiovascular mortality in population-based study and in patients with hypertension [27, 28]. In this experiment, HR in SHR CJID group was significantly decreased, suggesting that CJID is beneficial in inhibiting sympathetic nervous activity. Previous study identified oral administration of asiatic acid caused reduction of 32 mmHg SBP and HR simultaneously [28]. Therefore, CJID not only exhibited certain anti hypertension effect, but also decreased HR significantly, which is similar to the pharmacological mechanism of β-blocker. The result of our study demonstrated that CJID treatment prevented cardiac hypertrophy remodeling in SHRs. It has been determined that the response of increasing BP in the chronic hypertension, lead structural and functional changes specifically [10]. Our hypertensive rat model was characterized by left cardiac remodeling. Characteristics included elevation of LVMI, LV wall thickness which showed increase in the echocardiogram images, while left ventricular fractional shortening and ejection fraction were decreased. The histology observation identified cardiomyocytes diameter to be enlarged. The presence of LVH and cardiac dysfunction is a common complication of arterial hypertension, and as an indicator of end organ damage. These means, both BP lowering and LVH regression were accompanied by favorable outcome in the present study. It has been characterized that SHRs persisted and developed into high BP stage, and caused left ventricle impaired generating LVH compared with the normotensive WKY [29].

It is well known that hypertension is associated with ROS. Given evidence suggested that redox-dependent pathways significantly contribute to the pathophysiology in hypertension [30] and high level of ROS play a role in the pathophysiology of hypertensive cardiac remodeling [31]. A major feature of ROS-induced cellular injury is lipid peroxidation due to the effect of ROS on polyunsaturated fatty acid [32]. It results in the disruption of cell’s membrane lipid bilayer arrangement and produces unsaturated aldehyde, malondialdehyde (MDA) [33]. These metabolites are capable of inactivating many cellular proteins by forming protein cross-linkages and causing cytotoxic effect [33]. In our experiment, there was a significant increase in MDA levels of SHRs’ serum and left ventricular tissue, while CJID treatment decreased its level in both tissues. Otherwise, intracellular enzymatic mechanism, superoxide dismutase (SOD), catalyzes the dismutation of the O_2_^∙^ free radical in water and hydrogen peroxide in serum and left ventricular tissue [34]. This study shows that hypertension caused SOD activity and its expression decreased in those tissues. Given off, CJID increased SOD activity and expression. This up regulation of SOD alleviates oxidative stress in SHRs and prevents further cardiovascular damage [35]. By contrast, SHRs were characterized by increased DHE and DCFH-DA fluorescence intensity in cardiac left ventricle. Treatment of CJID caused the intensity to decrease significantly which means CJID inhibits oxidative stress. Several cardiac NAD(P)H oxidase comprising NOX1, NOX2, and NOX4, appears to be required for the oxidative mechanisms involved in the development of left ventricular hypertrophy in hypertension. Therefore, alteration in ROS bioavailability by decreasing production and/or by increasing radical scavenging are potential therapeutic modalities to reduce blood pressure in hypertension and prevent left ventricular hypertrophy. Besides ROS generating enzymes, antioxidant defense systems are important for the oxidative stress that tremendously results. The intervention of hypertension aims not only by improving blood pressure, but also by mitigating oxidative stress in individuals with high blood pressure proving more effective at reducing cardiovascular disease risk.

To elucidate the mechanism of elevated levels of ROS, ROS are stimulated by major ROS-generating enzymes in cardiovascular system, NADPH oxidases (NOX) homologues, which we observed in western blotting method. Hypertensive rats were characterized by increased expressions in NOX1, NOX2 and NOX4. Previous studies denoted that the NOX1, NOX2, and NOX4 enzymes are expressed in cardiovascular tissues and not only participate in normal vascular and cardiac function but also contribute to the development of cardiovascular disease. NOX enzymes have very specific mechanisms and functions which are found in endothelial cells, vascular smooth muscle cells (VSMCs), and cardiomyocytes. Hypertension, a factor contributing to cardiovascular implications, acutely or chronically leads to cardiac upregulation. Several studies have shown that their expressions were elevated both in cardiomyocytes and vascular wall in a few *in vivo* animal models of hypertension [13, 36]. In the present study, we determined that CJID administration inhibited NOX1, NOX2 and NOX4 expressions.

The increased superoxide generation may in turn call for the increase in SOD activity to facilitate the conversion of superoxide to hydrogen peroxide [37]. The SOD expressions identify physiologic response enzymes, SOD2 (mitochondrial, MnSOD) and SOD3 (extracellular, EcSOD) serving as oxidative stress blockade [38]. Hypertensive state was identified by lower expressions of those enzymes. CJID increased the production of SOD in LV tissue. Higher amounts of endogenous antioxidant enzymes in LV tissue and the subsequent reduction of cardiac oxidative stress might explain the antihypertensive and antihypertrophic characteristics of CJID.

Emerging evidence supports the concept that hypertension may increase circulating pro-inflammatory mediators that trigger oxidative stress. NADPH oxidases control oxidative stress response that they serve as an oxygen sensor to generate ROS from molecular oxygen. Up-regulation of cardiac NOXs-induced by hypertension will further trigger inflammation. It has been reported that hypertension stimulates NF-κB (nuclear factor kappa B) activation [36]. To determine the role of NF-κB in the anti-inflammatory effects of CJID, we further determined its effect on NOXs-mediated left ventricular NF-κB translocation in SHRs. As shown in Supplementary Figure 3, we found that CJID administration inhibited left ventricular NF-κB translocation in SHRs. Therefore, these results provide additional evidence for the role of NF-κB in anti-inflammatory mechanisms of CJID in SHRs.

Our present study demonstrated that SHR treated with a daily dose of CJID over a period of 5 weeks demonstrated not only lower SBP and HR, but also the prevention of target organ damage in hypertension. In this respect, Indonesian folk medicine CJI, might inhibit left ventricular hypertrophy and left ventricular dysfunction through the mechanism of NADPH oxidases yielding oxidative stress formation. We can outline the following conclusions from the current study: (a) CJID treatment could reduce BP and HR, and suppress LVH remodeling in SHRs; (b) CJID treatment can alleviate NOX-based ROS generation and the expression of cardiac NOX1, NOX2, and NOX4; and (c) CJID-induced increased expressions of certain endogenous antioxidants SOD2 and SOD3 in the cardiac left ventricle were identified.

In conclusion, CJID inhibits cardiac hypertrophy through a mechanism that may involve the reduction of oxidative stress formation yielded by NADPH oxidase pathway. CJID treatment decreased BP and HR efficiently and reversed ventricular remodeling in SHRs. The mechanism is possibly related with the inhibition effect of CJID in the formation of ROS through cardiac NADPH oxidase pathway. This study also indicates improvement of cardiac endogenous antioxidant SOD by the administration of CJID. Therefore, our findings delivered a theoretical basis for using CJID in the treatment of hypertension and its myocardial hypertrophy. CJID might be a new candidate for cardioprotective drugs for patients with hypertensive vascular disease. These findings should be important for the advance in preclinical and clinical research.

## MATERIALS AND METHODS

### Composition of CJID

**Figure 9:**
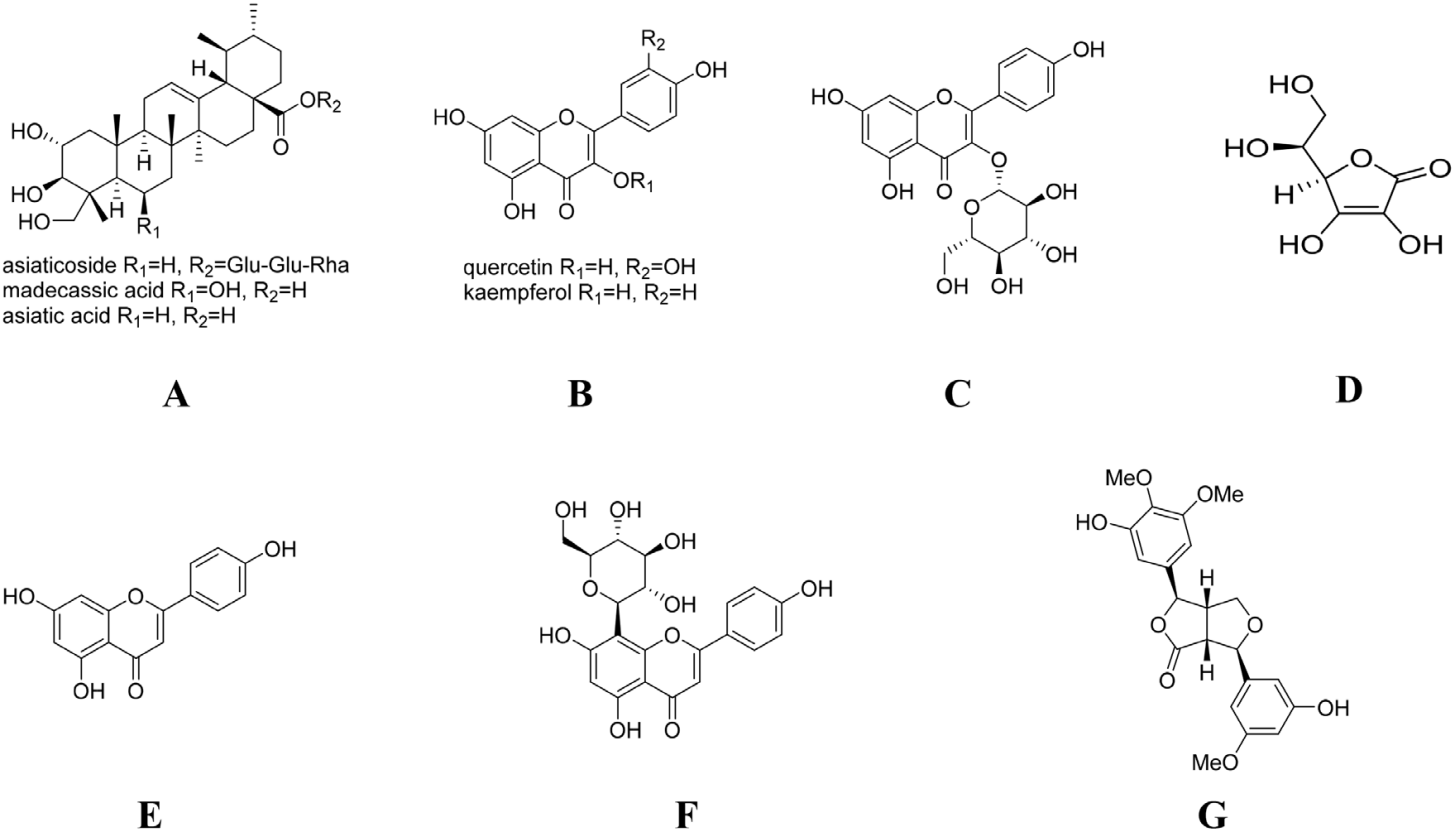
Chemical structure of the main ingredients of CJID **(A)** triterpenoids (asiaticoside, asiatic acid, and madecassic acid) of *C asiatica* and asiaticoside of *I. cylindrica*, **(B)** flavonoids (quercetin, kaempferol and astragalin) of *C asiatica, J. gendarussa* and *I. cylindrica*, **(C)** flavonoids (astragalin) of *C asiatica, J. gendarussa* and *I. cylindrica*, **(D)** ascorbic acid of *J. gendarussa*, **(E)** flavonoids (apigenin) of *J. gendarussa*, **(F)** flavonoids (vitexin) of *J. gendarussa*, and **(G)** lignans (*graminone B*) of *I. cylindrica.*

CJID consists of 3 herbs as shown in Table 2. The main chemical components of CJID is described in Figure 9. All herbs in dry form were provided and identified by Research and Development of Medicinal Plant and Traditional Medicine (B2P2TOOT), Ministry of Health, Indonesia (document number YK.01.03/2/615/2017). The powdered CJI stored at 4°C, were dissolved in distilled water and boiled in 90°C for 30 minutes prior to use.

### Experimental animals

All animal experimental procedures were performed in accordance to the guidelines of Taiwan legislations on the ethical use and care of laboratory animals. The study protocol was approved by the Animal Review Board of the Kaohsiung Medical University, Kaohsiung, Taiwan. Forty healthy male SHRs and their normotensive controls, WKY rats, at 8 weeks of ages, weighing 180 - 200 g, were purchased from National Laboratory Animal Breeding and Research Center, Taipei, Taiwan. All rats were housed in an animal facility with a 12 - hour light/dark cycle, provided standard food and drinking water ad libitum. Ambient temperatures were between 18°C and 22°C with humidity 45 - 70%.

### Measurement of SBP and HR

SBP and HR of all rats were weekly measured (6 times totally) using non-invasive tail-cuff manometer-tachometer (NATSUME KN-210, Natsume Seisakusho Co., Tokyo, Japan). In brief, conscious rats were placed in a restrainer (at temperature 29 ± 1°C for 10 - 15 minutes) and allowed to calm prior to blood pressure measurement. The rat tail was placed inside the tail cuff, and the cuff was automatically inflated and released. For each rat, blood pressure was recorded as the mean value from the three measurements with 15 minutes intervals.

### Measurement of cardiac hypertrophy

After this period, all rats were sacrificed with urethane anesthesia. Their hearts were removed immediately and washed with cold normal saline, then dried with filter paper. The heart length and width were measured and their pictures taken as well. The left ventricle was separated and its mass was accurately weighed by electronic balance. Then, LVMI was measured by calculating the ratio of left ventricular mass (mg) and body weight (g).

### Histological examination

Left ventricle was placed in 25 mM KCl & PBS relaxing buffer, then stored in 40% neutral-buffered formaldehyde at room temperature. Hematoxylin and eosin (H&E) stained sections (∼5 μm) were prepared to measure cardiomyocytes diameter at the papillary level [39]; so as to compare the cardiac dimensions at the same level for each heart using standard procedure muscles. The sections were photographed at 400x magnification using a Nikon Eclipse TE 2000-S (Tokyo, Japan) equipped with Nikon DS Fi1 digital camera and NIS-Element F software. Microscope lighting, focus, and field selection were optimized for distinction of cell boundaries. In order to increase the probability of cardiomyocytes being visualized in appropriate cross-section and not tangentially, fields were chosen so as to include the papillary muscles and the vertically aligned muscle layers adjacent to them. The cardiomyocytes were outlined manually and bucket-filled-in tiff. Images were opened in Image J and after setting the threshold, analyzed. Major and minor transverse axes and cross-sectional area were measured, and cardiomyocytes with an aspect ratio of ≥ 1.2 were excluded. This helped eliminate cells sectioned tangentially. Data from all the fields were combined and analyzed cells sectioned tangentially. Data from all the fields were combined and then analyzed.

### Measurement of left ventricular function

Left ventricular morphology and function were assessed by trans-thoracic echocardiography. All rats were anesthetized with 1% sodium pentobarbital and were fixed on the board. The ventral chest and front area hairs of rats were removed. The echocardiography probe emits and receives ultrasound in a range of 5 - 12 MHz. M-mode left ventricular end-systolic dimensions (LVESDs, millimeter) and left ventricular end-diastolic dimensions (LVEDDs, millimeter) were averaged from 3 - 5 beats. The fractional shortening (FS) and ejection fraction (EF) were calculated using the formula as follows:

$$FS=\left(\frac{LVEDD-LVESD}{LVEDD}\right)\times 100\%$$ (1)

[40]

$$EF=\left(\frac{LVEDD^{3}-LVEDS^{3}}{LVEDD^{3}}\right)\times 100\%$$ (2)

[40]

FS represents the percentage of left ventricle changes in cavity dimensions at the base with systolic contraction. EF represents global left ventricular function by assessing changes in the volume of the left ventricle during systolic contraction.

### Assays of MDA and SOD

Malondialdehyde in the serum and heart tissues were determined using a kit purchased from Sigma-Aldrich (St. Louis, USA; cat. no. MAK085) according to the manufacturer’s instructions. The activity of SOD was determined using the SOD assay kit (St. Louis, USA cat. no. KT-034). The MDA and SOD data were measured using the V-5100H spectrophotometer (BioTek Instruments, Inc., VT, USA).

### Detection of superoxide generation in the left ventricle

Dihydroethidium staining (DHE, Invitrogen, USA) was used to detect the *in situ* levels of superoxide (O_2_^∙−^) in the myocardium [41]. DHE is freely permeable to cells and in the presence of superoxide is oxidized to fluorescent ethidium bromide which is trapped intracellular by intercalation into the DNA. The left ventricular frozen sections (∼6 μm) on polylisine glass slides were incubated with 10 μM DHE at 37°C for 30 min in a humidified chamber protected from light. Fluorescent images of ethidium bromide were obtained using confocal laser-scanning microscope (Zeiss LSM 700, Carl Zeiss MicroImaging GmbH, Jena, Germany) and the number of nuclei labelled by DHE was automatically counted in each field with an image-analysis software (Zeiss-Axio vision software). The mean intensity fluorescence of myocytes nuclei was expressed as the fluorescence value of all the pixels in six randomly selected LV fields divided by the total number of pixels, obtained by confocal microscopy with identical laser and photomultiplier settings.

### Detection of H_2_O_2_ in the left ventricle

H_2_O_2_was determined with 2′, 7′-dichloro-dihydrofluorescein diacetate (DCFH-DA, Invitrogen, USA), a cell-permeable non-fluorescent probe which is de-esterified intracellularly and then oxidised to fluorescent 2′, 7′-dehydrofluorescin (DCF) upon oxidation. This is a validated method for the quantification of ROS in isolated myocytes [42]. Tissue was loaded with 10 μM DCFH-DA, obtained from a 2 mM stock solution of DCFH-DA prepared in ethanol, for 30 minutes at 37°C in the dark. Levels of DCF in tissue, sampled randomly in each preparation, were measured with laser scanning confocal microscope (200x magnification).

### Western blotting analysis

Frozen left ventricular tissue homogenates were weighed and diced into small pieces in ice-cold RIPA lysis buffer mixed with 1% TritonX100. RIPA lysis buffer content was 25 mM Tris-HCl pH 7.6, 150 mM NaCl, 1% NP-40, 1% sodium deoxycholate, 0.1% SDS (M-PER^®^, Thermo Scientific, USA; cat. no. 78501) with freshly added 1 mM (3.7 mg/tablet for 100 ml RIPA buffer) Phosphatase Inhibitor Cocktail tablets (cOmplete, Mini, Roche, Germany; cat. no. 11836153001). After sonication, the homogenates were centrifuged at 13,000 g for 30 minutes at 4°C, and the supernatant was recovered as the total cellular protein. Total protein from each sample was separated by sodium dodecyl sulfate polyacrylamide gel electrophoresis on 10% acrylamide gels, transferred to a polyvinylidine difluoride plus membrane, and then blocked with 5% nonfat dry milk in Tris-buffered saline. The membrane was subsequently incubated with a 1:1000 dilution of antibodies. These are NOX1 (MOX1 (H-75); Santa Cruz Biotech, USA; cat. no. sc-25545), NOX2 (Purified Mouse Anti - gp91 [phox]; BD Bioscience USA; cat. no. 611415), NOX4 (NOX4 (L-20); Santa Cruz Biotech, USA; cat. no. sc-55142), SOD2 (D3X8F; Cell signaling Tech, USA cat. no.13141) and SOD3 (Anti-Superoxide Dismutase 3 antibody; Abcam USA; cat. no. ab83108). Proteins were detected with horseradish peroxidase-conjugated secondary antibody (1:1000) dilution, chemicon, Tamecula, CA, USA). The immunoreactive bands were detected by chemiluminescence reagents developed by Hyperfilm (Kodak, Rochester, NY, USA), and the loading control protein beta-actin was used in the analyses.

### Statistical analysis

Data is expressed as mean ± SEM. The data was statistically evaluated using one-way analysis of variance (ANOVA), and a post hoc analysis was performed using Fisher’s least significant difference (LSD) test. *P* value less than 0.05 was considered to be statistically significant.

## SUPPLEMENTARY MATERIALS FIGURES AND TABLE


