# Supplementary material for: Indonesian herbal medicine prevents hypertension-induced left ventricular hypertrophy by diminishing NADPH oxidase-dependent oxidative stress
Source: Oncotarget. 2017 Sep 30;8(49):86784–98. doi: 10.18632/oncotarget.21424 (PMC5689725; doi:10.18632/oncotarget.21424)
Supplement: Supplementary file 1 [file oncotarget-08-86784-s001.pdf]

Indonesian herbal medicine prevents hypertension-induced left ventricular hypertrophy by diminishing NADPH oxidase-dependent oxidative stress

SUPPLEMENTARY METERIALS

Physiological variables

Every week food and water intake, urine and stool output were 24 hours observed. Samples collection obtained from each rats where it was placed in individual metabolism cage. Feed efficiency per kilocalories consumed calculated with the relationship below:

$$Feed\ efficiency\left(FE\right)=\frac{Weight\ gained\left(g\right)}{consumed\left(g\ kcal\right)}\times 100$$

where, the caloric content of feed in kcal/g was based on 3.573 kcal/g value for standard chow diet [1].

Analysis of liver function and lipid profiles

After sacrifice, blood samples were collected by left ventricular blood. Liver function was determined based on changes in plasma levels of glutamic-oxaloacetic transaminase (GOT) and glutamic-pyruvic transaminase (GPT). Plasma levels of albumin, total protein, alkaline phosphatase (ALP), total cholesterol, triglyseride, low-density lipoprotein cholesterol (LDL-C), high-density lipoprotein cholesterol (HDL-C) were measured using Spotchem EZ SP 4430 (ARKRAY Inc, Kyoto, Japan).

Immunofluorescence assay of left ventricular NF-κB-p65 nuclear translocation

Immunofluorescence staining was according to the procedure as previously described [2]. Staining was performed on 5 µm acetone-fixed left ventricular frozen sections. The sections were incubated for 1 hour in 10% bovine serum to block non-specific protein-protein interactions. The sections were then incubated with primary anti-NF-kB-p65 antibody (Millipore, CA; cat. no. MAB3026) at a 1/100 dilution overnight at 4°C. Rhodamine (TRITC)-conjugated goat anti-mouse secondary antibody (Millipore, CA; cat. no. NG1929384) was used to reveal the staining. DAPI was used to stain the cell nuclei. Fluorescent pictures were photographed with Zeiss Axio Imager Z1 (Zeiss LSM 700, Carl Zeiss MicroImaging GmbH, Jena, Germany).

REFERENCES

1. Fraulob JC, Ogg-Diamantino R, Fernandes-Santos C, Aguila MB, Mandarim-de-Lacerda CA. A mouse model of metabolic syndrome: insulin resistance, fatty liver and non-alcoholic fatty pancreas disease (NAFPD) in C57BL/6 mice fed a high fat diet. J Clin Biochem Nut. 2010; 46:212-223.
2. Guo Y, Wang L, Ma R, Mu Q, Yu N, Zhang Y, Tang Y, Li Y, Jiang G, Zhao D, et al. JiangTang XiaoKe granule attenuates cathepsin K expression and improves IGF-1 expression in the bone of high fat diet induced KK-Ay diabetic mice. Life Sci. 2016; 148:24-30.

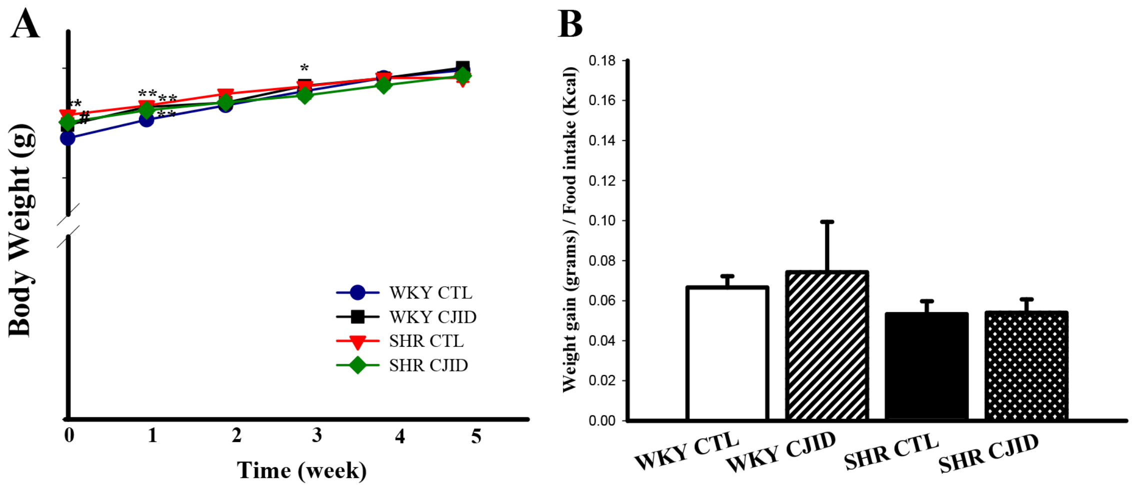

Supplementary Figure 1: Body weight evolution and weight gain. **(A)** Body weight evolution during five weeks of study. **(B)** Weight gain in each group. Each point represents the mean ± SEM. $n = 10$. *$P < 0.05$, **$P < 0.01$, *when compared to WKY CTL; $^{\#}P < 0.05$, $^{\#}$when compared to SHR CTL.

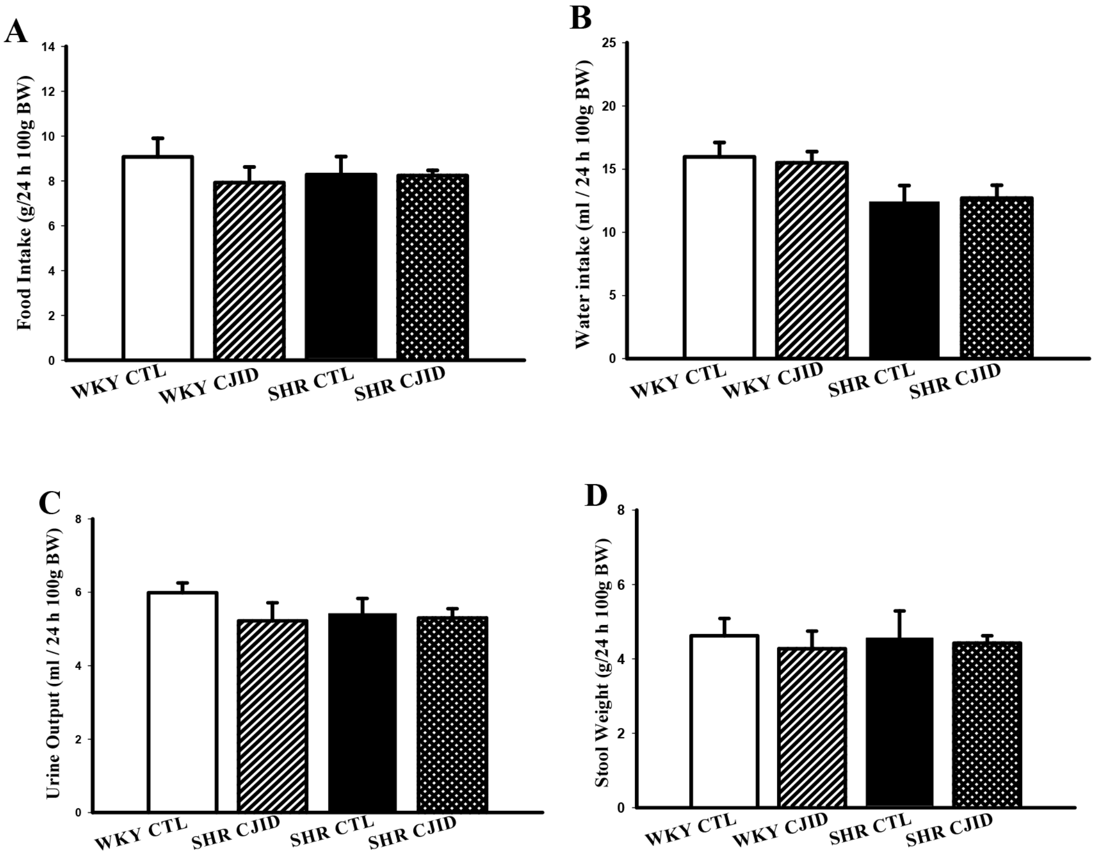

Supplementary Figure 2: Effects of CJID on cumulative food and water intake, urine volume and stool weight. **(A)** Food intake, **(B)** water intake, **(C)** urine output, **(D)** stool weight in each group. Each point represents the mean ± SEM. *n* = 10.

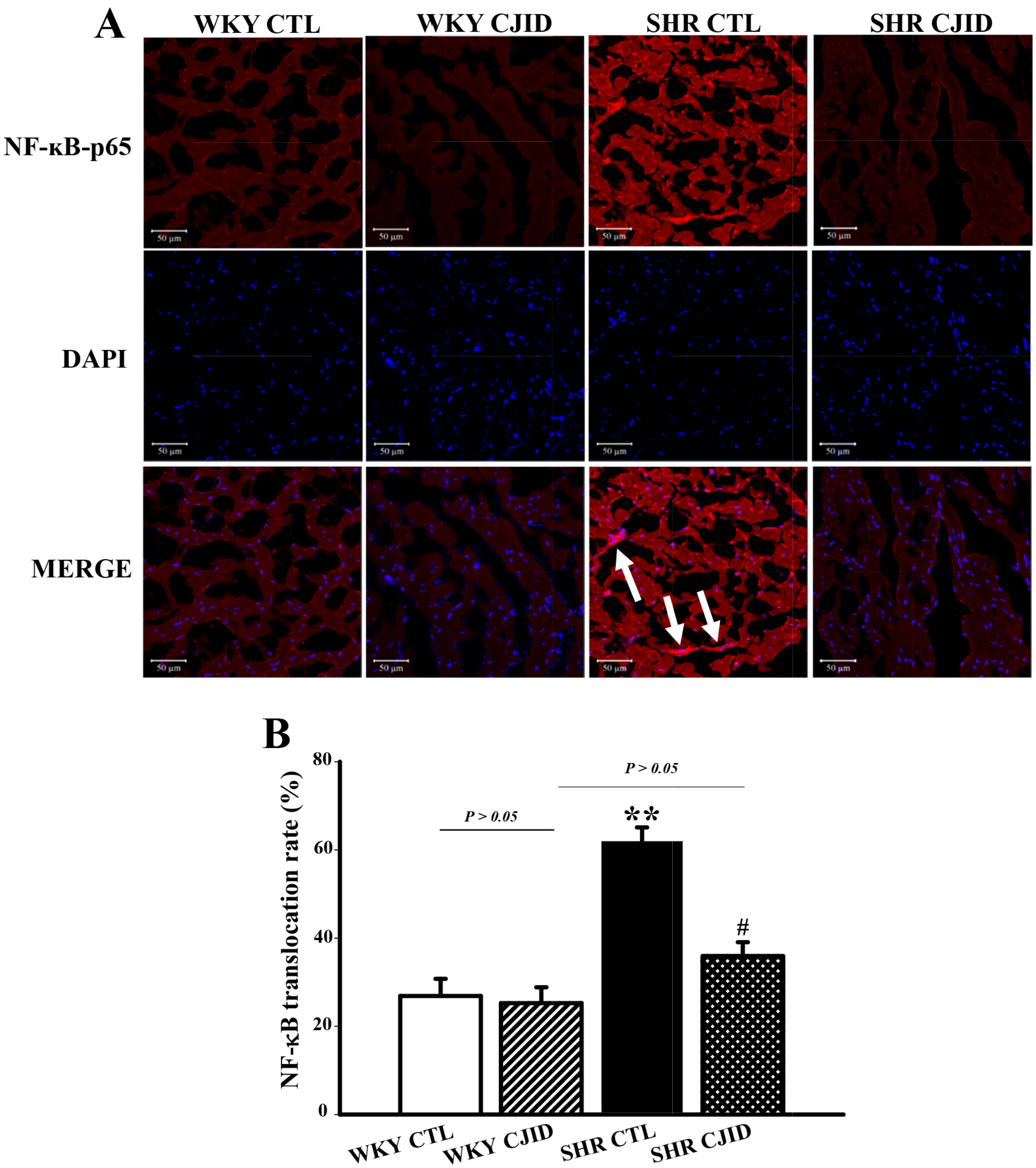

Supplementary Figure 3: Effects of CJID on left ventricular NF-κB-p65 nuclear translocation. (A) The representative confocal immunofluorescence staining observations (200x) of nuclear cardiomyocytes. Section were counterstained with rhodamine-labeled mouse antibody. Arrows show nuclear translocation. **(B)** NF-κB-p65 nuclear translocation in the left ventricle myocardium. The red color represent NF-κB-p65 staining, the blue color represent nuclei staining, and the magenta color (reddish-blue) represent nuclear translocation. Data are presented as mean ± SEM, n = 10. **P < 0.01, *when compared to WKY CTL; #P < 0.05, #when compared to SHR CTL.

Supplementary Table 1: Serum markers in liver function and lipid profile

Biochemical indices	WKY CTL	WKY CJID	SHR CTL	SHR CJID
**Liver function**				
GOT (IU/L)	37.2 ± 8.8	28.3 ± 3.5	31.8 ± 3.9	27.2 ± 4.7
GPT (IU/L)	33.4 ± 8.6	65.7 ± 10.2	42.5 ± 6.3	54.0 ± 8.4
Albumin (g/dL)	3.2 ± 0.2	3.7 ± 0.1*	3.7 ± 0.0*	3.7 ± 0.2
Total protein (g/dL)	6.3 ± 0.3	6.2 ± 0.2	6.1 ± 0.1	6.0 ± 0.3
ALP (IU/L)	116.7 ± 13.7	104.2 ± 7.3	133.0 ± 7.6	113.8 ± 9.6
**Lipid profile**				
Total cholesterol (mg/dL)	97.6 ± 7.5	99.5 ± 8.4	49.8 ± 1.7**	39.2 ± 6.6**
Triglyceride (mg/dL)	49.7 ± 10.6	102.0 ± 16.2	52.0 ± 13.8	23.7 ± 6.8
LDL-C (mg/dL)	66.7 ± 6.4	50.2 ± 8.1	32.6 ± 2.5**	31.1 ± 5.8**
HDL-C (mg/dL)	22.7 ± 1.5	28.8 ± 1.5	8.1 ± 1.2	12.0 ± 2.1^{NS}

Values represent Mean ± SEM, $n = 10$. ^{NS}not significant. *$P < 0.05$, **$P < 0.01$, *when compared to WKY CTL.
